# Regulation of anti-apoptotic Bcl-2 family protein Mcl-1 by S6 kinase 2

**DOI:** 10.1371/journal.pone.0173854

**Published:** 2017-03-16

**Authors:** Alakananda Basu, Savitha Sridharan

**Affiliations:** Institute for Molecular Medicine, University of North Texas Health Science Center, Fort Worth, Texas, United States of America; University of South Alabama Mitchell Cancer Institute, UNITED STATES

## Abstract

The anti-apoptotic Bcl-2 family protein myeloid cell leukemia-1 (Mcl-1) plays an important role in breast cancer cell survival and chemoresistance. We have previously shown that knockdown of the 40S ribosomal protein S6 kinase-2 (S6K2), which acts downstream of the mechanistic target of rapamycin complex 1 (mTORC1), enhanced breast cancer cell death by apoptotic stimuli. The increase in cell death by S6K2 depletion was partly due to inactivation of Akt. In the present study, we investigated if S6K2 regulates Mcl-1, which acts downstream of Akt. Silencing of S6K2 but not S6K1 in T47D cells decreased Mcl-1 level, and potentiated apoptosis induced by TRAIL and doxorubicin. Knockdown of S6K2 also decreased the level of anti-apoptotic Bcl-xl. Depletion of the tumor suppressor protein PDCD4 (programmed cell death 4), which regulates translation of several anti-apoptotic proteins, reversed downregulation of Bcl-xl but not Mcl-1 and failed to reverse the effect of S6K2 knockdown on potentiation of doxorubicin-induced apoptosis. Downregulation of Mcl-1 by S6K2 knockdown was partly restored by the proteasome inhibitor MG132. Overexpression of catalytically-active Akt or knockdown of glycogen synthase kinase-3 (GSK3)-β, a substrate for Akt, had little effect on Mcl-1 downregulation caused by S6K2 deficiency. Silencing of S6K2 increased the level of c-Jun N-terminal kinase (JNK) and knockdown of JNK1 increased basal Mcl-1 level and partly reversed the effect of S6K2 knockdown on Mcl-1 downregulation. JNK1 knockdown also had a modest effect in attenuating the increase in doxorubicin-induced apoptosis caused by S6K2 deficiency. These results suggest that S6K2 regulates apoptosis via multiple mechanisms, and involves both Akt and JNK.

## Introduction

Breast cancer is a heterogeneous disease with multiple factors contributing to the poor survival and the severity of the disease. The phosphatidylinositol 3-kinase (PI3K)/Akt/mechanistic target of the rapamycin (mTOR, previously known as mammalian target of rapamycin) pathway is deregulated in more than 50% of breast cancers [1–3]. mTOR interacts with either raptor or rictor and several other proteins to form complex 1 (mTORC1) or complex 2 (mTORC2), respectively [4]. The 40S ribosomal protein S6 kinases (S6Ks) act downstream of mTORC1 [4]. There are two S6K homologs, S6K1 and S6K2 [4]. We have previously shown that silencing of S6K2, but not S6K1, enhanced breast cancer cell death by apoptotic stimuli [5]. S6K2 was also shown to promote fibroblast growth factor-2 (FGF2)-mediated survival of small cell lung cancer (SCLC) cells [6].

The Bcl-2 family proteins play important roles in regulating cell death by apoptosis [7]. The Bcl-2 family consists of both anti-apoptotic (*e*.*g*., Bcl-2, Bcl-xl and Mcl-1) and pro-apoptotic (*e*.*g*., Bax, BAD, Bim and Bid) members [7]. Tumor suppressor proteins can regulate the balance between anti-apoptotic and pro-apoptotic Bcl-2 family proteins. For example, the pro-apoptotic Bax and Bid are transcriptional targets of p53 [8–10] whereas Bcl-2 is negatively regulated by p53 [11–13]. We have shown that depletion of S6K2 caused an increase in cell death via p53/Bid in MCF-7 breast cancer cells [5].

The anti-apoptotic Bcl-2 family member, myeloid cell leukemia 1 (Mcl-1) has been implicated in promoting survival of many cancers, including breast cancer [14, 15]. Mcl-1 has a short half-life and its level is highly regulated at multiple levels, including transcriptional, translational and post-translational processes [15, 16]. Both phosphorylation and ubiquitination regulate the stability of Mcl-1. Several oncogenic signaling pathways, including PI3K/Akt, mTOR and mitogen-activated protein kinase (MAPK) pathways were shown to upregulate Mcl-1 [15].

GSK3β is believed to be the primary kinase responsible for Mcl-1 degradation [17]. The c-Jun N-terminal protein kinase (JNK) was also shown to be required for Mcl-1 degradation since prior phosphorylation of Mcl-1 by JNK was required for its phosphorylation by GSK3 [18]. We have previously shown that S6K2 mediates its pro-survival signaling partly via Akt in breast cancer cells [5]. Since GSK3β is a substrate for Akt, which phosphorylates and inhibits it [17], we investigated if S6K2 regulates Mcl-1 degradation in breast cancer cells. We made a novel observation that S6K2 positively regulates Mcl-1 partly via JNK-dependent but GSK3-independent pathway.

## Materials and methods

### Materials

Goat polyclonal antibody against S6K2 was purchased from R&D Systems (Minneapolis, MN). Mouse monoclonal antibodies against actin and tubulin were purchased from Sigma-Aldrich (St Louis, MO). Monoclonal antibody against PARP was obtained from Pharmingen (San Diego, CA). Antibodies against S6K1 (rabbit monoclonal), cleaved caspase-3 (rabbit polyclonal), phospho-S6 (rabbit polyclonal), T389S6K (mouse monoclonal), PDCD4 (rabbit monoclonal), Mcl-1 (rabbit polyclonal), JNK (mouse monoclonal), Akt (rabbit polyclonal), phospho-Akt (Ser473, rabbit monoclonal and mouse monoclonal), phospho-GSK3β (Ser9, rabbit monoclonal), phospho-Mcl-1 (Ser159/Thr163, rabbit polyclonal), phospho-Mcl-1 (Thr163, rabbit monoclonal) and phospho-SAPK/JNK (Thr183/Tyr185, rabbit monoclonal) were purchased from Cell Signaling Technology (Danvers, MA). Polyclonal antibody against Mcl-1 and JNK, and monoclonal antibodies against GSK3α/β, Mcl-1 and Bcl-xl were purchased from Santa Cruz Biotechnology (Dallas, TX). Lys48-specific anti-ubiquitin antibody (rabbit monoclonal) was purchased from EMD Millipore (Bedford, MA). Horseradish peroxidase-conjugated donkey anti-rabbit, goat anti-mouse, and mouse anti-goat antibodies were obtained from Jackson Immuno Research Lab. Inc. (West Grove, PA). Polyvinylidene difluoride membrane was from Millipore (Bedford, MA) and enhanced chemiluminescence detection kit was from Perkin-Elmer (Shelton, CT). Control non-targeting and target-specific siRNAs were obtained from GE Dharmacon (Chicago, IL). Lipofectamine RNAiMax transfection reagent was obtained from Invitrogen, (Carlsbad, CA). Protease inhibitor and phosphatase inhibitor cocktails and MG132 were purchased from Calbiochem/EMD-Millipore (Bedford, MA).

### Cell culture

T47D and HCC1428 (ATCC) cells were maintained in RPMI 1640 medium supplemented with 10% fetal bovine serum and 2 mM glutamine. Cells were kept in a humidified incubator at 37°C with 95% air and 5% CO_2_. The cells were authenticated by DNA fingerprinting at the Department of Molecular & Medical Genetics at the UNT Health Science Center.

### Transfection

Cells were transfected with 10 to 20 nM control non-targeting or target-specific siRNAs using Lipofectamine RNAiMax transfection reagent by reverse transfection protocol. Briefly, siRNAs and Lipofectamine RNAiMax were diluted in 100 μl Opti-MEM medium without serum, mixed gently and incubated in the wells of the tissue culture plate for 10–20 min at room temperature. Approximately 1.2 to 1.5 x 10^5^ cells in complete growth medium were added to the siRNA-Lipofectamine RNAiMAX complex, rocked gently and incubated for 48 h to 72 h. Unless otherwise mentioned, cells were transfected with SMARTpool siRNA. The extent of gene knockdown was determined by Western blot analysis. Cells were infected with adenovirus vector containing GFP or constitutively-active (myrisotylated) Akt (MOI 10).

### Clonogenic cell survival assay

Cells transfected with or without control non-targeting or S6K2 siRNA were cultured until there were at least 50 cells per colony. At the end of the incubation, the cells were washed with PBS, fixed with methanol and incubated with 0.025% crystal violet solution in methanol for 15 min. Colonies were counted using ImageJ software (NIH) and the plate was photographed using the BioChemi System (BioImaging System, UVP, Upland, CA).

### Immunoblot analysis

Cells were harvested by trypsinization, washed twice with PBS and lysed in buffer containing 20 mM Tris-HCl, pH 7.4, 0.15 M NaCl, 1 mM EGTA, 1 mM EDTA, 1.0% Triton X-100, 0.5% Nonidet-40, 10 mM β-glycerophosphate, protease inhibitor cocktail and phosphatase inhibitor cocktail. Equivalent amounts of total proteins (20–25 μg) were electrophoresed by SDS-PAGE and transferred electrophoretically to polyvinylidene difluoride membrane (EMD Millipore, Bedford, MA). Immunoblot analyses were performed with 1:1,000 dilution of various antibodies (except actin, tubulin, PARP and GSK3 antibodies were used at 1:5,000 dilution) as described before [19]. The blots were visualized using the enhanced chemiluminescence detection reagents and the manufacturer’s protocol. The intensities of immunoreactive proteins were quantified using ImageJ software. The blots were probed with actin or tubulin to control for equal loading.

### Immunoprecipitation

Cells were lysed as described above, sonicated for 10 second and centrifuged at 10,000*g* for 15 min. The supernatant was collected, an aliquot was saved as input and 250 μg of protein was added to 1 μg monoclonal Mcl-1 antibody conjugated with protein A/G agarose beads and incubated overnight at 4°C. The immunoprecipitates were washed with TPBS and subjected to Western blot analysis.

### Statistical analysis

Statistical significance was determined by paired Student’s t-test using Microsoft Excel. A *p*-value < 0.05 was considered statistically significant.

## Results

### Knockdown of S6K2 decreased breast cancer cell survival

**Fig 1:**
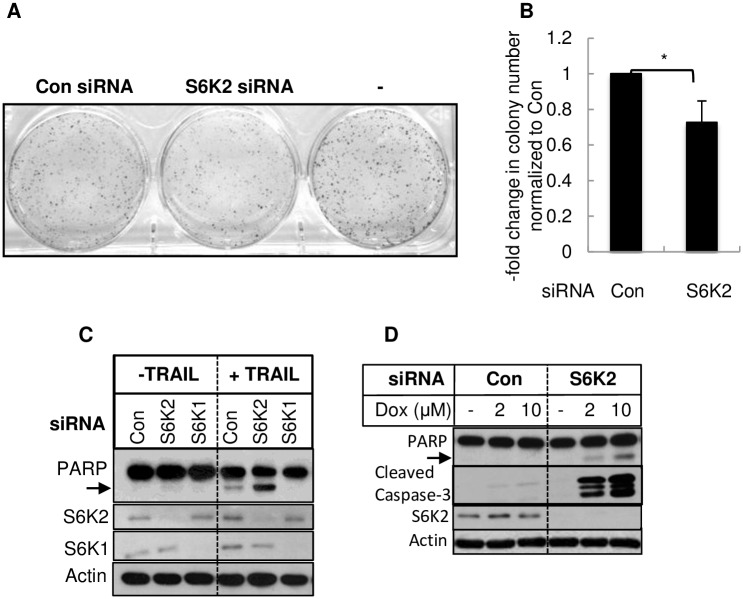
Knockdown of S6K2 decreased survival of T47D cells. Cells were transfected with or without control non-targeting siRNA or S6K2 siRNA. (A). Clonogenic assay was performed as described under “Materials and Methods.” (B). The bar graph represents average colonies ± SD of triplicate plates. *, *p*<0.05. (C). T47D cells were transfected with indicated siRNAs. 48 h after transfection, cells were treated with 0.25 nM TRAIL for 24 h, and Western blot analysis was performed with indicated antibodies. (D). T47D cells were transfected with indicated siRNAs. 48 h after transfection, cells were treated with indicated concentrations of doxorubicin for 24 h and Western blot analysis was performed with indicated antibodies. Actin was used as a loading control.

We have previously shown that knockdown of S6K2 enhanced cell death in MCF-7 and ZR75-1 breast cancer cells that contain wild-type p53 [5]. In the present study, we examined the effect of S6K2 knockdown on the survival of T47D breast cancer cells, which contain mutant p53. Fig 1 shows that knockdown of S6K2 caused a modest (Fig 1A) but significant (Fig 1B) decrease in the clonogenic survival of T47D breast cancer cells. Moreover, knockdown of S6K2 but not S6K1 enhanced apoptosis induced by TRAIL (Fig 1C) and doxorubicin (Fig 1D) as evidenced by the increase in the cleavage of PARP and the generation of cleaved caspase-3.

### Knockdown of S6K2 decreased anti-apoptotic Mcl-1

**Fig 2:**
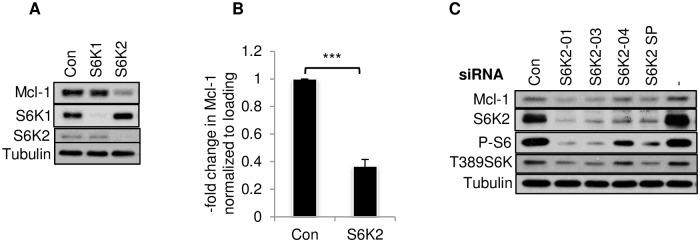
Knockdown of S6K2 decreased Mcl-1. T47D cells were transfected with indicated siRNAs as described under “Materials and Methods.” (A). Western blot analysis was performed with indicated antibodies. Tubulin was used as a loading control. (B). The intensity of Mcl-1 following transfection with SMARTpool (SP)-S6K2 siRNA, which is a combination of four individual siRNAs, was determined using ImageJ. Each bar represents mean ± S.E. of 8 independent experiments. ***, *p*< 0.0001 using paired Student’s t test. (C). T47D cells were transfected with or without indicated individual or SP siRNAs. Western blot analysis was performed with indicated antibodies. The results are representative of at least 3 independent experiments.

Bcl-2 family proteins were shown to be important regulators of S6K2-mediated cell survival [5, 20]. Since Bcl-2 is not detectable in T47D cells, we examined the effect of S6K2 knockdown on the anti-apoptotic Bcl-2 family protein Mcl-1. As shown in Fig 2A, knockdown of S6K2 but not S6K1 caused a substantial decrease in Mcl-1 protein level, and the decrease in Mcl-1 by S6K2 siRNA was statistically significant (Fig 2B). We then compared the effects of several individual S6K2 siRNAs as well as SMARTpool (SP) S6K2 siRNA, which is a combination of the individual siRNAs, on Mcl-1 level (Fig 2C). S6K2-01 and -03 siRNAs were most effective in decreasing S6K2 level, its phosphorylation status and phosphorylation of its substrate S6 [21, 22] when compared with both untransfected and non-targeting control siRNA-transfected T47D cells whereas S6K2-04 siRNA was least effective. Thus, there was a strong correlation between decrease in S6K2 level/activity and downregulation of Mcl-1.

### S6K2 knockdown enhanced proteasomal degradation of Mcl-1

**Fig 3:**
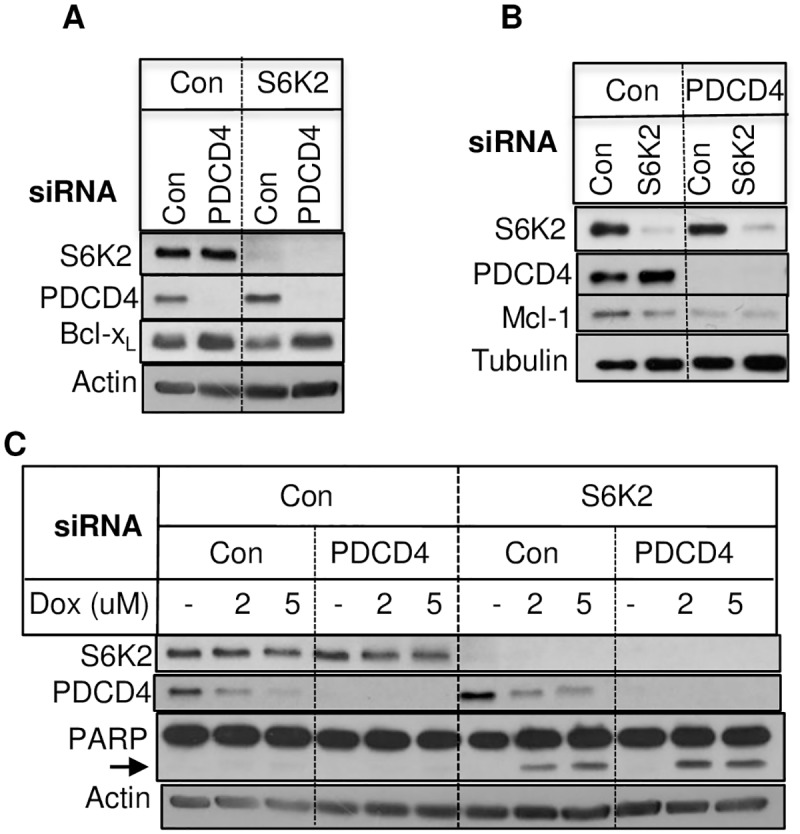
The effect of PDCD4 knockdown on anti-apoptotic Bcl-2 family proteins. (A & B). T47D cells were transfected with either control non-targeting siRNA, S6K2 siRNA, PDCD4 siRNA or co-transfected with both S6K2 and PDCD4 siRNA, and Western blot analyses were performed. (C). Cells transfected with indicated siRNAs for 72 h were treated with or without indicated concentrations of doxorubicin for 18 h and then Western blot analyses were performed. The arrow indicates cleaved PARP.

It has been reported that S6K2 regulates translation of anti-apoptotic Bcl-xl and XIAP via proteasomal degradation of the tumor suppressor protein PDCD4 [23, 24]. Therefore, we examined if S6K2 regulates Mcl-1 level via PDCD4. Fig 3A shows that knockdown of S6K2 also caused a decrease in Bcl-xl. While knockdown of PDCD4 reversed downregulation of Bcl-xl caused by S6K2 deficiency, silencing of PDCD4 failed to restore Mcl-1 level (Fig 3B). Moreover, PDCD4 knockdown had little effect on doxorubicin-induced PARP cleavage and it failed to reverse the effect of S6K2 knockdown in potentiating doxorubicin-induced PARP cleavage. These results suggest that PDCD4 is not responsible for downregulation of Mcl-1 or potentiation of cell death caused by S6K2 deficiency in T47D breast cancer cells.

**Fig 4:**
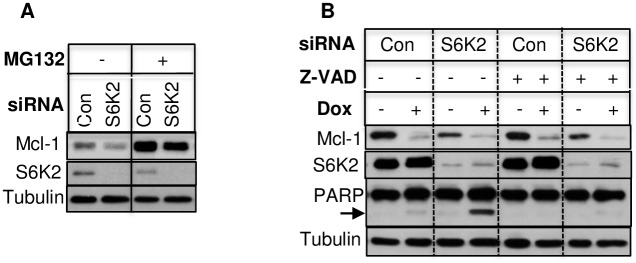
Effects of proteasome inhibitor and caspase inhibitor on Mcl-1 downregulation. (A). T47D cells were transfected with indicated siRNAs. 48 h after transfection, cells were treated with or without 5 μM MG132 for 24 h and Western blot analysis was performed. (B). T47D cells transfected with indicated siRNAs for 48 h were pretreated with or without 25 μM z-VAD-fmk prior to treatment with 10 μM doxorubicin for 24 h, and Western blot analysis was performed. Tubulin was used to check for equal loading.

Mcl-1 is regulated at the post-translational level via proteasome-mediated degradation [16] or caspase-mediated cleavage [25, 26]. The proteasome inhibitor MG132 increased basal Mcl-1 level and partly reversed the effect of S6K2 knockdown on Mcl-1 downregulation (Fig 4A). The caspase-inhibitor z-VAD was effective in inhibiting doxorubicin-induced apoptosis but it had little effect on S6K2 knockdown-mediated downregulation of Mcl-1 (Fig 4B). These results suggest that S6K2 knockdown enhances degradation of Mcl-1 via caspase-independent but proteasome-mediated pathway.

### Downregulation of Mcl-1 by S6K2 knockdown involved JNK but not GSK3

**Fig 5:**
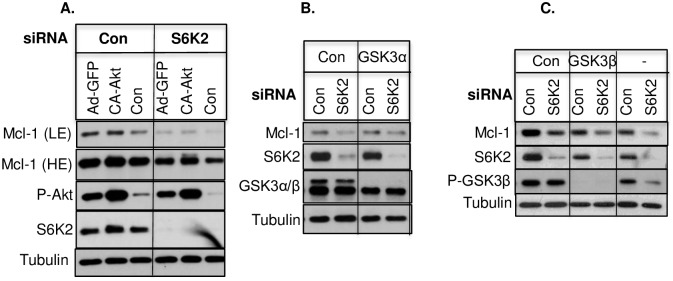
S6K2 knockdown-mediated downregulation of Mcl-1 does not involve Akt/GSK3. (A). T47D cells were infected with or without adenoviral vector containing GFP or CA-Akt construct. 48 h after transfection, cells were transfected with control non-targeting or S6K2 siRNA. Approximately, 72 h after siRNA transfection, cells were processed for Western blot analysis using indicated antibodies. (B and C). T47D cells were transfected with or without control non-targeting, GSK3α (B) or GSK3β siRNA (C) for approximately 72 h and Western blot analysis was performed. Results are representative of 3 independent experiments. LE, low exposure; HE-high exposure.

We have previously shown that S6K2 promotes breast cancer cell survival partly via Akt (5). Since GSK3 is a substrate for Akt and phosphorylation of Mcl-1 by GSK3 promotes degradation of Mcl-1 via the ubiquitin proteasome-mediated pathway [16, 17], we examined if S6K2 regulates Mcl-1 level via Akt/GSK3 pathway [16, 17]. As shown in Fig 5A, overexpression of catalytically-active (CA) Akt caused a slight increase in Mcl-1 but failed to prevent Mcl-1 downregulation caused by S6K2 deficiency. In addition, silencing of either GSK3α (Fig 5B) or GSK3β (Fig 5C) had little effect on S6K2 knockdown-mediated downregulation of Mcl-1.

**Fig 6:**
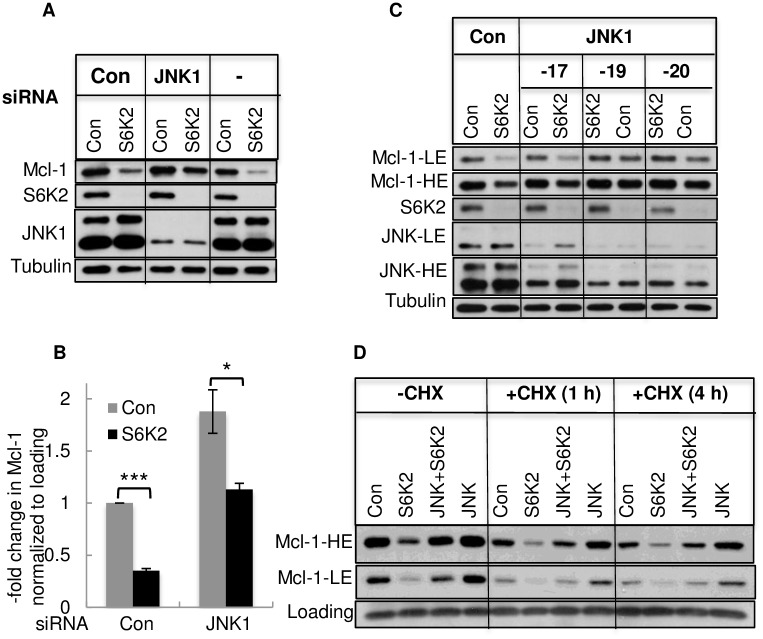
Effects of JNK1 knockdown on S6K2 knockdown-mediated downregulation of Mcl-1. (A). Cells were transfected with or without control or JNK1 siRNA and Western blot analysis was performed after 72 h. (B). The intensity of Mcl-1 was determined by ImageJ. Each bar represents mean ± S.E. of 6 independent experiments.***,*p*<0.0001; *, *p*<0.05 using paired Student’s t test. (C). Cells were transfected with control non-targeting or individual JNK1 siRNAs for approximately 72 h and Western blot analysis was performed. (D). Cells treated with indicated siRNAs for 48 h were then treated with 10 μg/ml cycloheximide for 1 h or 4 h, and Western blot analysis was performed.

Several studies suggested that the JNK signaling pathway regulates Mcl-1 level [16, 18, 27, 28]. We therefore, examined if knockdown of JNK1 prevents downregulation of Mcl-1 caused by S6K2 deficiency. As shown in Fig 6A, silencing of JNK1 increased basal Mcl-1 level and partially rescued the effect of S6K2 knockdown on Mcl-1 downregulation. Based on several independent experiments, knockdown of S6K2 decreased Mcl-1 by approximately 2.9-fold (1 vs 0.35) (Fig 6B). While knockdown of JNK1 increased Mcl-1 by approximately 1.9-fold (1 vs 1.88) when compared with control non-targeting siRNA-transfected cells, it increased Mcl-1 by 5.3-fold (0.35 vs 1.88) when compared with S6K2 siRNA-transfected cells. The combined knockdown of JNK1 and S6K2 attenuated S6K2-mediated downregulation of Mcl-1 from 2.8-fold (1 vs 0.35) to 1.7-fold (1.88 vs 1.13). We also examined the effects of several different JNK1 siRNAs for their ability to reverse Mcl-1 downregulation caused by S6K2 deficiency. Fig 6C shows that JNK1-19 and -20 siRNAs were more effective in knocking down JNK1 and reversing S6K2 knockdown-mediated downregulation of Mcl-1 compared to JNK1-17 siRNA. Thus, the extent of JNK1 knockdown correlated with its ability to reverse S6K2 knockdown-mediated downregulation of Mcl-1.

To determine if JNK1 knockdown restores Mcl-1 level by enhancing Mcl-1 translation, we examined if inhibition of Mcl-1 translation by the new protein synthesis inhibitor cycloheximide abrogates the effects of JNK1 knockdown on Mcl-1 level. Fig 6D shows that treatment of T47D cells with cycloheximide attenuated Mcl-1 level. Knockdown of S6K2 decreased Mcl-1 level which was partly restored by JNK1 knockdown even when new protein synthesis was blocked by cycloheximide. These results suggest that JNK1 knockdown prevents the degradation of existing Mcl-1.

**Fig 7:**
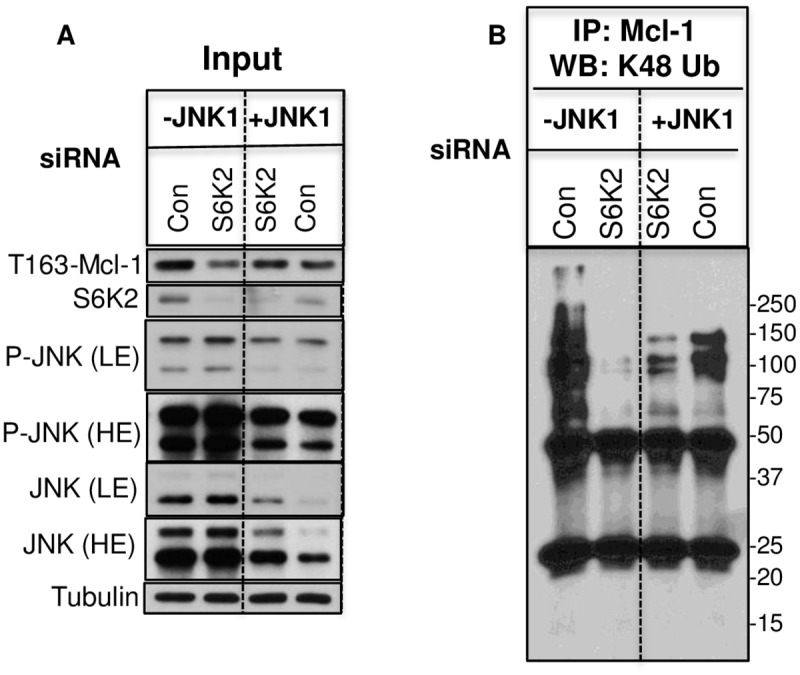
Effects of JNK1 and S6K2 knockdown on Mcl-1 phosphorylation and ubiquitination. Cells were treated with indicated siRNAs for 48 h and then treated with 10 μM MG132 for 6 h. Cells were then processed for immunoprecipitation using Mcl-1 antibody as described under “Materials and Methods.” Both inputs and immunoprecipitates were subjected to Western blot analysis using indicated antibodies.

Since phosphorylation of Mcl-1 at Ser159/Thr163 site can lead to its ubiquitination and proteasomal degradation [17], we examined the effects of S6K2 and JNK1 knockdown on Mcl-1 phosphorylation and ubiquitination. It was difficult to detect phosphorylated Mcl-1 unless cells were treated with MG132. Fig 7A shows that knockdown of S6K2 decreased Mcl-1 phosphorylation and this was associated with decrease in ubiquitinated Mcl-1 (Fig 7B). Knockdown of JNK1 restored both phosphorylated and ubiqutinated Mcl-1, suggesting that JNK1 regulates ubiquitin-mediated degradation of Mcl-1.

### Knockdown of JNK1 partly reversed the effect of S6K2 knockdown on apoptosis

**Fig 8:**
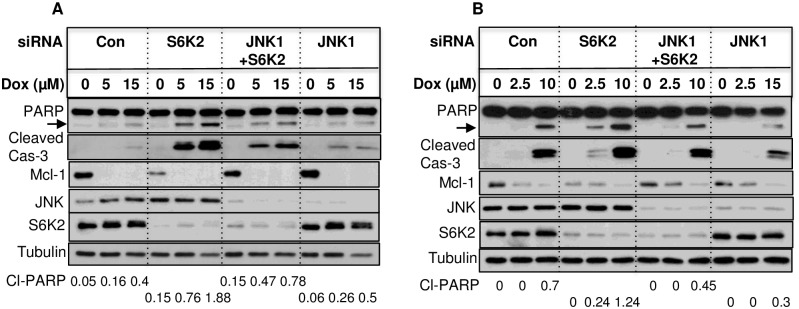
Knockdown of JNK1 attenuates increase in doxorubicin-induced apoptosis caused by S6K2 knockdown. T47D (A) or HCC1428 (B) cells were transfected with indicated siRNAs for approximately 48 h and then treated with or without doxorubicin for approximately 24 h. Western blot analysis was performed with indicated antibodies. The arrow indicates cleaved PARP. Results are representative of 2 (HCC1428) to 3 (T47D) independent experiments. The bands corresponding to cleaved PARP were quantified using ImageJ and the intensities of cleaved PARP normalized with tubulin are shown.

Since knockdown of S6K2 potentiates cell death by apoptotic stimuli, we examined if JNK1 knockdown mitigates the increase in apoptosis caused by S6K2 deficiency. As shown in Fig 8A, treatment of T47D cells with doxorubicin had little effect on the cleavage of PARP or pro-caspase-3 when cells were transfected with either control, S6K2 or JNK1 siRNA. While S6K2 knockdown enhanced PARP cleavage and the generation of cleaved caspase-3, combined knockdown of JNK1 and S6K2 counteracted the effect of S6K2 knockdown on doxorubicin-induced apoptosis. We quantified cleavage of PARP using ImageJ software. As shown in Fig 8A, S6K2 knockdown increased the abundance of cleaved PARP following treatment with doxorubicin but the effect was reduced when JNK1 and S6K2 were both depleted. To determine if the effect of S6K2 knockdown on Mcl-1 level is unique to T47D cells, we extended our study to another breast cancer cell line HCC1428, which also lacks wild-type p53. As shown in Fig 8B, knockdown of S6K2 also decreased Mcl-1 and enhanced doxorubicin-induced cleavage of PARP and pro-caspase-3. Knockdown of JNK1 partly restored Mcl-1 level and reversed the effect of S6K2 knockdown on doxorubicin-induced cleavage of PARP and pro-caspase-3. These results suggest that JNK1 knockdown attenuates but does not prevent the effect of S6K2 knockdown on doxorubicin-induced apoptosis.

## Discussion

We have previously shown that S6K2 promotes breast cancer cell survival via p53-dependent pathway [5]. The results of our present study show that S6K2 also promotes survival of breast cancer cells that contain mutant p53. We made a novel observation that S6K2 positively regulates the level of the anti-apoptotic Bcl-2 family protein Mcl-1. We further demonstrate that S6K2 regulates Mcl-1 partly via the JNK pathway.

Mcl-1 has a short half-life, and its level is controlled at the transcriptional, post-transcriptional, translational and post-translational level [15]. Based on qPCR analysis, knockdown of S6K2 either had no effect or caused a small decrease in *Mcl-1* mRNA. The mTOR signaling pathway plays an important role in protein translation. It has been reported that S6K2 may regulate cap-independent translation of selective mRNAs, such as Bcl-xl, Mcl-1 and XIAP mRNAs, through the presence of internal ribosome entry site (IRES) at their 5’UTR [29]. The tumor suppressor protein PDCD4 (programmed cell death 4) binds to the IRES to repress translation [24]. PDCD4 is a substrate for S6K2 and phosphorylation of PDCD4 triggers its degradation via the ubiquitin proteasome-mediated pathway [24]. In fact, S6K2 was shown to regulate translation of Bcl-xl and XIAP via PDCD4 [24] and promote FGF2-mediated survival of SCLC cells via proteasomal degradation of PDCD4 causing increased translation of XIAP and Bcl-xl [20]. Consistent with the published reports, S6K2 knockdown also caused downregulation of Bcl-xl, and this was blocked by knockdown of PDCD4 (Fig 3A). However, PDCD4 knockdown failed to restore Mcl-1 level (Fig 3B).

Although both S6K1 and S6K2 act downstream of mTORC1, we have shown that the two homologs have distinct effects on breast cancer cell survival (Fig 2) [5]. In the present study, we show that knockdown of S6K2 but not S6K1 caused downregulation of Mcl-1 (Fig 2C). It is well established that inhibition of mTORC1/S6K1 leads to activation of Akt involving a negative feedback loop [30–32]. We have previously shown that in contrast to S6K1, S6K2 positively regulates Akt and overexpression of constitutively-active Akt partially rescued the effect of S6K2 knockdown on cell death [5], suggesting that Akt may be partly responsible for the survival effect of S6K2. Similar results on Akt were obtained in S6K2 knock-out T cells compared to wild-type cells [33]. Thus, the differential effects of the two homologs on cell death could be explained by their differential effects on Akt activity.

The PI3K/Akt pathway also plays an important role in the regulation of Mcl-1. Akt phosphorylates and inhibits GSK3β, which is believed to be the primary kinase that regulates Mcl-1 stability [17]. Mcl-1 phosphorylated by GSK3 is recognized by the ubiquitin proteasome system and is targeted for degradation [16, 17]. We found that the proteasome inhibitor MG132 increased Mcl-1 and partly restored the effect of S6K2 knockdown on the downregulation of Mcl-1 (Fig 4A). Therefore, we hypothesized that S6K2 regulates Mcl-1 at the post-translational level via the Akt/GSK3 pathway. However, overexpression of Akt (Fig 5A) or knockdown of GSK3α (Fig 5B) or GSK3β (Fig 5C) had little effect in reversing the effect of S6K2 knockdown on Mcl-1 downregulation. Thus, downregulation of Mcl-1 by S6K2 may not involve the Akt/GSK3 pathway.

The stability of Mcl-1 is also regulated by the MAPK pathway [15]. While phosphorylation of Mcl-1 by ERK stabilizes it, phosphorylation of JNK may either stabilize or degrade Mcl-1 depending on the cellular context [16]. Our results show that JNK, in fact, negatively regulates Mcl-1 in both T47D and HCC1428 breast cancer cells, and knockdown of JNK1 partially reversed Mcl-1 downregulation caused by S6K2 deficiency (Fig 6A, 6B & 6C). It has been reported that phosphorylation of Mcl-1 at Thr163 by JNK primes phosphorylation of Mcl-1 at Ser159 by GSK3, and production of a phosphodegron that targets Mcl-1 for ubiquitin-mediated degradation [18, 27]. However, in contrast to normal cells where JNK-induced phosphorylation of Mcl-1 at Thr163 targets it for degradation via the GSK3 pathway, Mcl-1 is not degraded via this pathway in cancer cells [27]. Thus, our results are consistent with the observation made by Nifoussi *et al*. [27]. We could detect phosphorylated Mcl-1 only when its degradation was inhibited by MG132. While knockdown of S6K2 decreased phosphorylation of Mcl-1 at Thr163, knockdown of JNK1 restored Thr163 phosphorylated Mcl-1. It is conceivable that when Mcl-1 degradation was inhibited by MG132, it also caused accumulation of Mcl-1 phosphorylated at T163 by ERK. We have, however, found that MG132 or JNK1 knockdown failed to completely reverse S6K2 knockdown-mediated downregulation of Mcl-1. This may be because of incomplete knockdown of S6K2 or JNK1 siRNA. In addition, multiple mechanisms may be involved in S6K2-mediated downregulation of Mcl-1. For example, mTOR signaling pathway was shown to regulate cap-dependent protein translation of Mcl-1 [34]. Future studies should determine if S6K2 also increases Mcl-1 by enhancing its translation.

Mcl-1 is an important anti-apoptotic protein that promotes survival of many cancers and confers resistance to therapeutic agents [15, 16, 27]. The expression of Mcl-1 was associated with poor prognosis of breast cancer patients [35]. To determine the functional significance of S6K2/JNK-mediated Mcl-1 regulation, we determined if restoration of Mcl-1 level by JNK1 knockdown has any impact on increase in apoptosis caused by S6K2 knockdown. We have shown that knockdown of S6K2 alone had only modest effect on breast cancer cell survival but it enhanced cell death by apoptotic stimuli (Figs 1 and 8) [5]. Although JNK1 knockdown did not prevent cell death, it did suppress the effect of S6K2 knockdown on doxorubicin-induced apoptosis in both T47D and HCC1428 cells (Fig 8A & 8B). The effect of JNK1 knockdown in reversing S6K2 knockdown-mediated apoptosis was, however, modest but was consistent with its partial ability to restore Mcl-1 level. JNK1 knockdown may also attenuate S6K2 knockdown-mediated apoptosis by inhibiting cell proliferation. In addition, since S6K2 regulates other Bcl-2 family members, such as Bcl-xl (Fig 3A), and promotes cell survival via Akt-dependent mechanisms [5], disruption of a single pathway may not be adequate to reverse its effect. Thus, our study suggests that S6K2 may engage multiple signaling pathways to promote breast cancer cell survival, including Akt and JNK1.
